# Pre-Operative Assessment of Patients with Cirrhosis for Extrahepatic Surgery

**DOI:** 10.1007/s11901-025-00697-4

**Published:** 2025-06-04

**Authors:** Claire Durkin, Nadim Mahmud

**Affiliations:** 1 Department of Medicine, Division of Gastroenterology and Hepatology, Perelman School of Medicine, University of Pennsylvania, Philadelphia, PA USA; 2 Department of Biostatistics, Epidemiology, and Informatics, Center for Clinical Epidemiology and Biostatistics, University of Pennsylvania, Philadelphia, PA USA; 3 Section of Gastroenterology and Hepatology, Corporal Michael J. Crescenz VA Medical Center, Philadelphia, PA USA; 4 Hospital of the University of Pennsylvania, 3400 Spruce Street, Philadelphia, PA 19104 USA

**Keywords:** Surgery, Cirrhosis, VOCAL-penn score, Mayo risk score, Model for end-stage liver disease, Child-turcott-pugh

## Abstract

**Purpose of Review:**

Patients with cirrhosis are at increased risk of peri-operative morbidity and mortality compared to those without cirrhosis, requiring careful pre-operative assessment of their liver disease, extra-hepatic comorbidities, and surgery-specific risk factors.

**Recent Findings:**

Adverse surgical outcomes in this population are often related to complications of advanced liver disease, including portal hypertension, impaired hemostasis, malnutrition/sarcopenia, and infection. Risk prediction tools, including the Child-Turcotte-Pugh score, Model for End-Stage Liver Disease score, Mayo Risk Score, and VOCAL-Penn Score, can be used to estimate post-operative mortality and support clinical decision-making when assessing surgical candidacy. Several common procedures, including hernia repair, laparoscopic cholecystectomy, and sleeve gastrectomy, are well-tolerated in appropriate candidates. Pre-procedural transplant evaluation and referral to a high-volume cirrhosis surgery center should be considered when feasible.

**Summary:**

This review discusses the pathophysiological mechanisms underlying increased peri-operative risk in cirrhosis, the application of surgical risk scores, liver-related contraindications to surgery, and specific considerations for several common procedures.

## Introduction

Patients with cirrhosis face an increased risk of surgical and anesthesia-related complications compared to those without cirrhosis, including a threefold higher risk of peri-operative mortality [1]. Despite widespread recognition of these risks in patients with advanced liver disease, the volume of surgeries in this population is rising due to the increasing prevalence of cirrhosis and an aging demographic [2, 3]. Pre-operative assessment in patients with cirrhosis requires careful consideration of liver disease severity and symptoms, including recognition of portal hypertension, impaired synthetic dysfynction, sarcopenia and malnutrition, and altered hemostasis. In addition, it is essential to understand non-hepatic medical comorbidities and surgery-specific factors as part of the pre-operative evaluation. Several risk prediction models, including the Child-Turcotte-Pugh (CTP) score, Model for End-Stage Liver Disease (MELD) score, Mayo Risk Score (MRS), and VOCAL-Penn Score (VPS) are used to estimate post-operative mortality and can inform clinical judgement when weighing the risk-benefit of a procedure. Given these complexities, a thorough and individualized pre-operative assessment is key to selecting appropriate surgical candidates, optimizing outcomes, and minimizing peri-operative complications in the setting of cirrhosis.

### Human and Animal Rights

All reported studies/experiments with human or animal subjects performed by the authors have been previously published and complied with all applicable ethical standards (including the Helsinki declaration and its amendments, institutional/national research committee standards, and international/national/institutional guidelines).

### Risk Factors for Surgical Complications

**Fig. 1:**
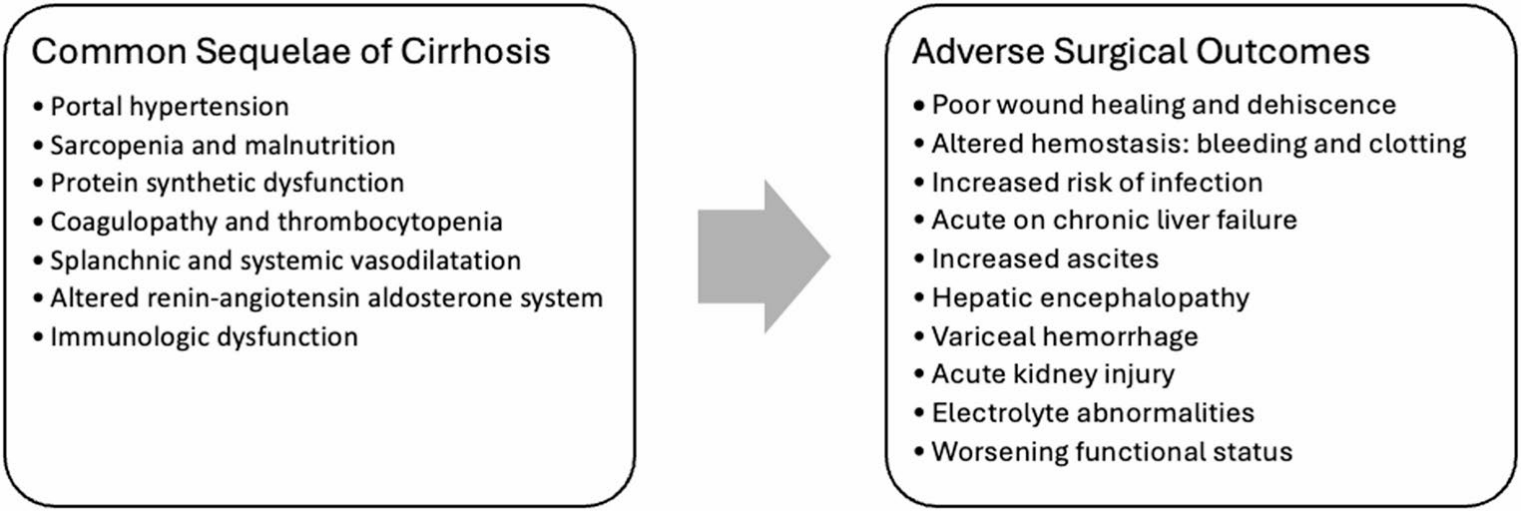
Common sequelae of cirrhosis and impacts on post-operative adverse outcomes

The increased risk of post-operative adverse outcomes in patients with cirrhosis can be attributed to several pathophysiological mechanisms outlined in Fig. 1. Several key risk factors are discussed below.

#### Portal Hypertension

Portal hypertension is a common manifestation of cirrhosis, arising from increased intrahepatic vascular resistance due to structural changes from liver fibrosis, elevated vascular tone in the hepatic microcirculation, and hyperdynamic circulatory changes [4]. Clinically significant portal hypertension (CSPH) is defined by a hepatic venous pressure gradient (HVPG) ≥ 10mmHg [4]. Due to increased risk of operative mortality with increasing HPVG, consensus guidelines have concluded that patients with an HPVG ≥ 16mmHg and especially ≥ 20mmHg are at elevated risk of undergoing surgery (HR > 2.5 and HR = 5.67 for 1-year mortality, respectively; *p* < 0.001) [5, 6]. Complications of portal hypertension include ascites, the pathologic accumlation of fluid within the peritoneal cavity, and varices, the portosystemic collateralization of blood vessels. In patients with cirrhosis, surgery has been implicated in the development and worsening of hepatic decompensations, including ascites, hepatic encephalopathy, and variceal bleeding [7]. One large, multicenter study demonstrated that approximately 9% of patients with cirrhosis developed a hepatic decompensation post-operatively [7]. The mechanism by which decompensations develop is multifactorial. General anasthesia reduces the arterial blood flow to the liver predisposing it to ischemic injury, which can be exacerbated by splanchnic bed manipulation during surgery, increased intra-abdominal pressure from positive pressure ventilation or laparoscopic approaches, and vasoactive medications [1]. Ascites may worsen or newly develop peri-operatively due to resuscitation with fluids and blood products, as well as the discontinuation of home diuretics [1]. The presence of ascites also can restrict pulmonary function and increase the risk of aspiration during induction of anesthesia [8]. Additionally, ascites predisposes patients to poor abdominal wound healing, where increased intra-abdominal pressure, fluid leaks, and associated edema increase the risk of wound dehiscence, infection, and hernia [9]. In patients with gastroesophageal varices, surgery has been hypothesized to increase the risk of variceal rupture and bleeding due to hymodynamic shifts during surgery resulting in expansion of portosystemic collaterals and increased hydrostatic pressure [1]. 

#### Bleeding and Clotting

Patients with cirrhosis are at elevated risk for both hemorrhage and thrombosis due to complex pathophysiological changes in primary hemostasis, coagulation, and fibrinolysis [10]. The risk of bleeding is largely driven by decreased synthesis of clotting factors (e.g., prothrombin and factors V, VII, IX, and X), along with thrombocytopenia and platelet dysfunction. In contrast, the risk of clotting is influenced by reduced synthesis of anticoagulant proteins (e.g., protein C, protein S, antithrombin), increased production of prothrombotic factors (e.g., fibrinogen, Von Willebrand factor, factor VIII), impaired fibrinolysis, endothelial dysfunction, and altered blood flow [10]. Among patients with cirrhosis admitted for non-surgical procedures, approximately 7% experience bleeding, including 2.3% with major bleeding [11]. While data are limited on bleeding following extrahepatic surgical procedures in the setting of cirrhosis, the risk of hemorrhage is estimated to be higher for more invasive procedures [12]. Among patients with chronic liver disease undergoing hepatectomy, risk factors for major bleeding include older age, presence of CSPH, hepatitis C and alcohol-associated liver disease, elevated creatinine, and use of certain medications (e.g., anticoagulants, antiplatelets, or non-steroidal anti-inflammatory drugs) [13, 14]. In addition to increased bleeding risk, studies also show that venous thromboembolism is common in patients with chronic liver disease, occuring in up to 8% of hospitalized patients [15]. Deep vein thrombosis, pulmonary embolism, and portal vein thrombosis all occur more frequently in patients with cirrhosis compared to those without [15]. Associated with increased morbidity and mortality, peri-operative bleeding and thromboembolism are major complications of surgery [10]. 

#### Malnutrition, Frailty, and Sarcopenia

Sarcopenia and frailty are common manifestations of cirrhosis characterized by loss of muscle mass and function [16]. They frequently develop in advanced liver disease due to a combination of imparied protein synthesis and a catabolic state, where energy needs exceed intake, leading to accelerated muscle breakdown [16]. In addition, patients with cirrhosis often experience malnutrition as a result of decreased nutrient intake and malabsorption [16]. Studies estimate that between 25 and 70% of patients with cirrhosis have sarcopenia and approximately 17–68% have frailty [16, 17]. Both conditions have been implicated in increased post-operative mortality and adverse outcomes, including delayed wound healing, increased hospital length of stay, and inpatient readmissions [18, 19]. In one large study, patients with cirrhosis and high risk frailty had a 74% increased hazard of post-operative mortality relative to low risk frailty patients [18]. Thus, as potentially modifiable risk factors for surgery, nutritional status and physical conditioning should be optimized pre-operatively to minimize surgery-related complicated and improve recovery. The mainstays of therapy for malnutrition, frailty, and sarcopenia are a combination of (1) nutritional support, focusing on adequate energy and protein intake, and (2) physical activity-based interventions, incorporating both aerobic and resistance exercises, and (3) medical optimization of cirrhosis, including addressing ascites and hepatic encephalopathy [16]. Treatment typically requires a multidisciplinary team consisting of a patient’s primary care provider, gastroenterologist/hepatologist, registered dietician, certified exercise physiologist/physical therapist, and/or a behavioral health specialist [16]. 

#### Infection

Patients with cirrhosis are an increased risk of developing infection as a result of cirrhosis-associated immune dysfunction, gut dysbiosis, increased bacterial translocation, and portosystemic shunting [20]. Studies have shown that infection increases mortality 4-fold among patients with cirrhosis and that approximately a third of hospitalized patients with cirrhosis develop bacterial infection [20]. Post-operative infections also are associated with an increased risk of mortality, including following bariatric and colorectal surgeries [21, 22]. A recent large, international, multicenter study demonstrated that 45% of hospitalized patients with cirrhosis who developed a bacterial or fungal infection underwent an invasive procedure in the previous month [23]. In this study, procedures were also significantly associated with infections from multi-drug resistant organisms (*p* < 0.001) [23]. 

### Surgical Risk Scores

Pre-operative assessment of surgical risk is important for understanding a patient’s potential for post-operative adverse outcomes and can help inform the decision whether to proceed with a procedure. Several tools routinely used in clinical practice to assess the severity of liver disease, including the MELD and CTP scores, can provide insight into a patient’s clinical status prior to a procedure, though they do not incorporate any surgery-specific risk factors [24–27]. Universal surgical risk calculators, such as the American College of Surgeons National Surgical Quality Improvement Program (ACS-NSQIP), are well validated to estimate post-operative mortality in surgical patients, but do not account for measures of liver dysfunction leading to an underestimate of risk in those with cirrhosis [28–30]. Two prediction models, the MRS and VPS, were developed specifically to aid in peri-operative risk stratification among patients with cirrhosis and attempt to address liver disease severity, non-hepatic comorbidities, and surgery-specific factors [31, 32]. Table 1 summarizes the variables comprising commonly utilized surgical risk scores.

**Table 1:** Components of cirrhosis surgical risk models

	CTP Score	MELD Score	Mayo Risk Score	VOCAL-Penn Score
Laboratory Components	- Bilirubin - Albumin - INR	- Bilirubin - Creatinine - INR - Sodium^*†^ - Albumin^†^	- Bilirubin - Creatinine - INR	- Albumin - Bilirubin - Platelet count
Clinical Factors	- Ascites - Encephalopathy	- Age^†^	- Age - Etiology of cirrhosis	- Age - BMI - MASLD - ASA Score
Surgical Considerations	-	-	-	- Type of surgery - Emergency vs. elective - Laparoscopic vs. open

Abbreviations: ASA = American Society for Anesthesiologists, BMI = body mass index, CTP = Child-Turcotte-Pugh, INR = international normalized ratio, MASLD = metabolic-associated steatotic liver disease, MELD = Model for End-Stage Liver Disease, VOCAL = Veterans Outcomes and Costs Associated with Liver Disease

^*^ Additional laboratory components incorporated as part of the MELD-Na score

^†^ Additional laboratory components and clinical factors incorporated as part of the MELD 3.0 score

It is important to remember that surgical risk stratification tools were derived and validated in patients considered to be surgical candidates and should be used in conjunction with clinical judgement. The decision to undergo surgery should involve shared decision-making with patients and a multidisciplinary team of liver and surgery specialists, weighing the risks and benefits of a procedure within the context of a patient’s medical history, individual circumstances, and personal preferences.

#### CTP and MELD Scores

The CTP uses 5 clinical and laboratory parameters—ascites, hepatic encephalopathy, international normalized ratio [INR], serum albumin, serum total bilirubin—to score the severity of liver disease on a scale from 5 to 15 and categorize patients into CTP class A, B, or C. Historically, the CTP score was developed to guide selection of candidates for surgery for portal hypertension (portocaval shunting and transection of the esophagus) and subsequently was utilized in liver transplant allocation [27, 33, 34]. More recently, it has been used as a global assessment of liver disease and prognosis.

The MELD score was originally developed to predict the 3-month survival of patients with cirrhosis undergoing a transjugular intrahepatic portosystemic shunt (TIPS) and was based on a patient’s serum total bilirubin, creatinine, and INR [35]. In 2002, it was adopted by the United Network for Organ Sharing (UNOS) to prioritize patients for liver transplant allocation after demonstrating the ability to accurately predict pre-transplant survival in end-stage liver disease [24, 36]. More recent iterations of the MELD score—the MELD-Na introduced in 2016 and MELD 3.0 in 2023—were developed to optimize waitlist mortality predictions and more equitably allocate organs to patients on the liver transplant waitlist, additionally incorporating serum sodium, serum albumin, and sex [26, 37]. 

Both the CTP and MELD scores have been evaluated as predictors of post-operative morbidity and mortality. However, as general models of liver function and disease severity, they do not account for the type or complexity of procedure, presence of extra-hepatic comorbidities, or overall functional status when evaluating surgical risk in patients with cirrhosis. Studies suggest that patients who have CTP A cirrhosis, low MELD scores (< 10), and/or no hepatic decompensations (i.e., no ascites, encephalopathy, or variceal bleeding) can safely undergo most surgeries, particularly if low risk and elective procedures [38–42]. For example, 90-day post-operative mortality following abdominal surgery for patients with cirrhosis with a MELD score < 10 was 1.7%, which was similar to the 1.2% mortality rate for patients without chronic liver disease [41]. Early studies also showed that patients with CTP A cirrhosis experienced 10% mortality following abdominal surgery, similar to contemporary mortality rates for patients without cirrhosis, and significantly higher than the 20–30% and 60–80% post-operative mortality experienced by those with CTP B and C cirrhosis, respectively [43–45]. 

#### Mayo Risk Score (MRS)

The MRS was the first dedicated surgical risk prediction model specifically developed to estimate post-operative mortality in patients with cirrhosis. Developed from a single-center cohort of patients with cirrhosis who underwent major cardiovascular, abdominal, and orthopedic surgeries from 1980 to 2004, the MRS incorporates age, American Society of Anesthesiologists (ASA) classification score (3 for compensated or 4 for decompensated cirrhosis), total bilirubin, creatinine, INR, and etiology of liver disease (alcohol-associated/cholestatic or viral/other) to compute probability of mortality following surgery [31]. The original MRS study found that patients with cirrhosis were at increased risk of mortality up to 90-days post-procedure and demonstrated a 30-day mortality ranging from 5.7% for those with low MELD scores (< 8) to more than 50% in those with elevated MELD scores (> 20) [31]. 

When externally validated in additional populations, the MRS has been found to overestimate surgical risk with inadequate calibration [30, 32, 46]. For example, among a Korean cohort of patients with cirrhosis, the predicted 1-year mortality rate using the MRS was significantly higher than what was observed (22.6% vs. 8.9%, respectively; *p* < 0.01). Similarly, a study of European patients with advanced chronic liver disease demonstrated that the median mortality risk predicted by the MRS at 30- and 90-days post-surgery was higher than predicted by other models including the VPS [30]. By overestimating surgical risk, patients who are at acceptable risk may be needlessly declined for an otherwise indicated surgical procedure. Additional criticisms of the MRS include that it was developed using a single-center cohort that may not adequately reflect institution-specific practices and the study cohort was restricted to those undergoing select surgeries [47]. Although the type of procedure performed is a major risk factor of post-operative mortality, it was not included as a predictor in the MRS [48]. Additionally, there have been significant developments in surgical techniques and peri-operative care since the MRS was developed in 2007, including increasing utilization of minimally invasive, endovascular, and robotic surgical approaches, likely contributing to the decreasing calibration of the MRS over time [32]. 

#### VOCAL-Penn Score (VPS)

The VPS is the most recently developed surgical risk stratification tool for patients with cirrhosis. Derived from a large, multicenter cohort of United States (U.S.) Veterans, it provides risk predictions for post-operative mortality 30-, 90-, and 180-days following extrahepatic surgery [32]. Similar to prior surgical risk models, it incorporates assessments of liver disease severity and markers of overall health; however, the VPS also includes type of surgery (i.e., open abdominal, laparoscopic abdominal, abdominal wall, vascular, major orthopedic, or chest/cardiac), as prior studies have demonstrated post-operative outcomes vary widely by procedural category [48]. Other predictors in the VPS include urgency of surgery, age, albumin, platelet count, total bilirubin, ASA class, body mass index (BMI), and presence of metabolic dysfunction-associated steatotic liver disease (MASLD) [32]. 

When internally validated within the Veterans Health Administration, the VPS demonstrated excellent discrimination (30-day post-operative mortality c-statistic = 0.859, 95% confidence interval [CI] 0.809–0.909) with a superior performance compared to the MELD, MELD-Na, CTP, and MRS at all evaluated timepoints [32]. When externally validated among 2 U.S. academic health systems and a Spanish cohort, the VPS continued to surpass other risk stratification models in terms of discrimination, calibration, and overall performance for determining mortality risk following extrahepatic surgery [30, 49]. Several studies also have validated additional applications of the VPS, including predicting 90-day post-operative decompensation in patients in patients with cirrhosis and post-operative mortality for liver resection [7, 50]. 

### Specific Populations

Several hepatic conditions are associated with significantly increased risk for peri-operative mortality and are generally considered contraindications to surgery.

#### Populations with Contraindications to Surgery

Acute liver failure (ALF), which is characterized by severe acute liver injury, hepatic synthetic dysfunction (INR ≥ 1.5), and altered mental status, carries a poor prognosis with a mortality rate as high as 85% without liver transplantation [51]. Patients with ALF generally require intensive care with complications including metabolic derangements, encephalopathy, cerebral edema, seizures, and renal failure and are unlikely to survive surgery outside of liver transplantation.

Alcohol-associated hepatitis is the acute onset of liver inflammation related to heavy alcohol use with a high risk of short-term mortality of 30–50% at 28-days [52]. The majority of data on peri-operative outcomes in this population are from the 1960–80s, when surgical diagnosis and intervention of alcohol-associated hepatitis were more common. Most studies demonstrate > 50% mortality in patients with alcohol-associated hepatitis following surgery including portosystemic shunting, exploratory laparotomy, and open liver biopsy [53–55]. However, one study found similar survival following portocaval shunt surgery between patients with alcohol-associated hepatitis and cirrhosis [56]. It is important to note that the operations studied are associated with high morbidity regardless of underlying alcohol-associated hepatitis and likely overestimate surgical risk of more modern, minimally invasive procedures in this population [57, 58]. Nevertheless, surgery should be approached with caution in the acute setting of alcohol-associated hepatitis. Additionally, because there is the possibility of hepatic recompensation with sustained alcohol abstinence, elective surgeries should be delayed to allow for possible resolution of hepatic decompensations and improvement in liver function.

Though data are limited, studies suggest elevated operative mortality in the setting of acute viral hepatitis. One study of patients with unspecified acute hepatitis demonstrated an 11.9% incidence of major complications and a 9.5% mortality rate in those with viral hepatitis following surgery [59]. 

### Common Surgeries

The operative risks associated with surgery vary widely depending on the type of surgery, as procedures can vary widely in complexity, invasiveness, location, duration, urgency, and recovery. Table 2 summaries the post-operative mortality by surgery category at 30-, 90-, and 180-days among U.S. Veterans in the VPS cohort [32]. In patients with cirrhosis, procedures associated with the highest risk of mortality include open abdominal, chest/cardiac, and major orthopedic surgeries, while laparoscopic abdominal and abdominal wall procedures were associated with relative low risk of mortality [3, 32, 48]. Considerations for several common surgeries in patients with cirrhosis are discussed below.

**Table 2:** Post-operative mortality by surgery category among U.S. Veterans

Surgery Category	30-Day Mortality	90-Day Mortality	180-Day Mortality
Abdominal - laparoscopic	0.63%	1.68%	2.52%
Abdominal - open	6.62%	11.58%	15.19%
Abdominal wall	1.61%	3.13%	5.20%
Vascular	1.82%	4.91%	7.09%
Major orthopedic	3.16%	6.70%	8.63%
Chest/cardiac	4.82%	8.67%	11.33%

*Adapted from Mahmud et al. 2021 [47] with permission

#### Hernia Repair

Abdominal wall hernias affect approximately 20–40% of patients with cirrhosis [60]. They often develop secondary to increases in intra-abdominal pressure created by ascites. If left unrepaired, abdominal wall defects increase the risk of bowel incarceration, strangulation, rupture, pressure necrosis and breakdown of overlying skin, ascitic leak, and peritonitis [61]. Medical management is often attempted prior to surgical repair with attempts to control patients’ underlying ascites with diuretics, paracentesis, and portosystemic shunting [61]. However, studies suggest that elective hernia repair can safely be performed in patients with cirrhosis with post-operative mortality rates of 0.6–1.2% in several large retrospective cohorts [42, 62], though patients with cirrhosis are at increased risk for mortality from hernia surgery compared to those without cirrhosis [42, 62, 63]. The decision of how to manage a hernia should be individualized, weighing the risk of surgery against the potential complications and symptoms associated with leaving a hernia intact (i.e., incarceration, spontaneous rupture, etc.). One randomized controlled trial demonstrated no difference in hernia-related complications or quality of life metrics between patients with umbilical hernias who underwent elective repair vs. conservative management, noting a 17% rate of recurrent hernia following surgery [42]. This study suggests that medical management may be an appropriate alternative to surgical repair of hernias, particularly in those felt high risk for surgery. Importantly, deferring elective hernia surgery can increase the risk of emergent repair, which carries significantly higher morbidity and mortality compared to elective repair in patients with cirrhosis [42, 60, 62–66]. For example, a recent meta-analysis demonstrated a significant higher odds of mortality among patients with cirrhosis after emergent vs. elective umbilical hernia surgery (OR = 2.67, 95% CI 1.87–3.97) [63]. There also may be missed opportunities for elective intervention, as about a third of patients who required emergent umbilical hernia repair were candidates for non-emergent repair in the prior year [42]. Concordant with these findings, a recent Markov decision analytic study demonstrated that patients with cirrhosis and abdominal hernia would be expected to benefit from elective hernia repair even up to a MELD-Na of 21 [67]. If repair is pursued, studies suggest that elective surgery should be preceded by optimization of ascites, nutritional status, liver disease, and medical comorbidities [42, 60, 62, 64]. 

#### Cholecystectomy

Cholelithiasis is more prevalent in patients with liver disease compared to the general population, affecting about 30% of those with cirrhosis [68]. The indications for cholecystectomy are generally the same for both patients with and without cirrhosis, including conditions such as acute cholecystitis, choledocholithiasis, gallstone pancreatitis, and biliary cholic [63]. Asymptomatic patients with gallstones, regardless of the population, are generally not recommended to undergo cholecystectomy [63]. When indicated, laparoscopic cholecystectomy is preferred over open cholecystectomy in patients with cirrhosis due to lower rates of mortality, post-operative complications, and hospital length of stay [69–72]. Studies have largely evaluated outcomes following laparoscopic cholecystectomy in patients with CTP A or B cirrhosis with low post-operative mortality rates of approximately 0.7–1.3% [70, 71], though patients with cirrhosis are still at increased risk of death following cholecystectomy compared to those without liver disease [72]. While data are limited in patients with CTP C cirrhosis, cholecystectomy is associated with high rates of mortality and surgical complications in this population [73]. Alternative therapies targeted at patients’ underlying condition should be explored in those with high risk for surgery, including medical management with antibiotics, percutaneous cholecystostomy or drainage, and endoscopic approaches, such as endoscopic retrograde cholangiopancreatography (ERCP) with sphincterotomy and balloon extraction and/or endoscopic ultrasound (EUS)-guided gallbladder drainage [74, 75]. It is important to note that patients with cirrhosis are also at elevated risk of complications from non-surgical alternatives. In one retrospective study of patients who underwent cholecystostomy, patients with cirrhosis were at elevated risk of post-operative complications such as bleeding (*p* < 0.001) compared to those without cirrhosis, but there was no difference in overall survival [76]. Similarly, studies have demonstrated increased risk of ERCP-related complications, particularly hemorrhage and post-ERCP pancreatitis, in patients with vs. without cirrhosis [77, 78]. 

#### Bariatric Surgery

Studies estimate that about 30% of patients with cirrhosis and over half of liver transplant recipients in the United States are obese with a body mass index ≥ 30 mg/kg^2^ [79, 80]. In patients with cirrhosis, obesity is a major risk factor for mortality, hepatic decompensation, hepatocellular carcinoma, portal vein thrombus, and acute-on-chronic liver failure [79]. Because weight loss is associated with clinical improvement in advanced liver disease, including regression of hepatic fibrosis and reduction in portal hypertension, treatment of obesity is a key component of disease management in cirrhosis [81, 82]. Obesity can be treated with lifestyle interventions, pharmacotherapy, and/or weight-loss procedures including bariatric surgery [83]. While many studies demonstrate significant weight loss with lifestyle modifications, these studies often evaluate intensive behavioral interventions by multidisciplinary teams that can be difficult to implement in clinical practice and participants frequently experience weight gain after study completion. Obesity guidelines have historically recommended approaching pharmacotherapies weight loss with caution in the setting of liver disease [83]. However, more recent data suggest that weight loss medications such as glucagon-like peptide-1 (GLP-1) receptor agonists can lead to improvements in obesity, hepatic steatosis, and liver biomarkers in those with MASLD [84]. 

Nevertheless, bariatric surgery has been shown to be safe and effective in well-selected candidates with advanced liver disease with a mortality rate of < 1% in patients with compensated cirrhosis [85]. It not only has been associated with weight loss, but also a reduction in liver-related adverse events, improvements in hepatic steatosis, and a decrease in cardiometabolic comorbidities (i.e., diabetes, hypertension, and dyslipidemia) [86, 87]. There are several different laparoscopic options for weight loss surgery, including roux-en-y gastric bypass (RYGB), sleeve gastrectomy, and gastric banding, each with distinct advantages and disadvantages. While RYGB typically results in the greatest weight loss among these procedures [88], sleeve gastrectomy is increasingly becoming the preferred option for bariatric surgery in patients with cirrhosis, as it is technically easier, requires shorter operating time, results in fewer vitamin deficiencies, and allows for endoscopic access to the stomach and biliary system in the event of complications, such as gastrointestinal bleeding or biliary obstruction [89, 90]. Multiple recent studies have demonstrated the safety and efficacy of laparoscopic sleeve gastrectomy in the setting of cirrhosis, including in patients awaiting liver transplantation [91–95]. Among patients with CTP A cirrhosis, rates of post-operative complications following sleeve gastrectomy are similar compared to those for patients without cirrhosis [91]. Although historically popular, gastric banding is becoming less common, as it is less effective for sustained weight loss compared to other bariatric surgeries and can result in complications such as band migration, erosion, perforation, and infection [96]. 

### Pre-Operative Referrals

Clinical practice guidelines can help guide peri-operative management of patients with cirrhosis, as well as the medical optimization of hepatic decompensations including ascites, bleeding, and encephalopathy before and after surgery [4, 8, 10, 97, 98]. In patients with high risk of clinical decompensation or in whom symptoms of portal hypertension are a significant barrier to surgery, evaluation for transplant evaluation and/or preemptive placement of TIPS can be considered.

#### Liver Transplant Evaluation

Because patients with cirrhosis may experience worsening liver function and hepatic decompensation following surgery, expert consensus recommends pre-operative evaluation for liver transplant when the 3-month post-operative mortality risk is > 15% or if the MELD score is > 15 [8]. These thresholds can help identify patients who are at high risk for post-operative complications and would benefit from consultation at a liver transplant center prior to surgery. While there may be practical limitations to transplant evaluation, such as the need for an emergent procedure or clear medical or psychosocial contraindications, early referral ensures multidisciplinary assessment, pre-operative optimization, and an understanding of transplant candidacy that can inform whether to proceed with an intervention.

#### Referral to High-Volume Cirrhosis Surgery Centers

Even if pre-operative liver transplantation evaluation is not pursued, patients with cirrhosis being considered for major elective surgery should preferentially undergo surgery at experienced centers. Though data are limited, a recent study of 14,500 surgeries in patients with cirrhosis across 110 centers in the Veterans Health Administration demonstrated that high cirrhosis surgical volume centers (> 16 surgeries in the year prior to index surgery) had a 36% reduced hazard of post-operative mortality through 90 days relative to low-volume centers (HR 0.64, 95% CI 0.43–0.94, *p* = 0.02) [99]. Further studies are needed to identify specific center practices that translate to reductions in operative risk, however this likely includes specialization of anesthetic management, operative technique, and post-operative monitoring and early intervention for complications.

#### TIPS Evaluation

Portal hypertension and its clinical manifestations are associated with increased risks of post-operative hepatic decompensation and mortality [5, 7, 32]. A TIPS addresses portal hypertension by creating a shunt between the portal and hepatic vein [100]. Because it can improve ascites, decompress varices, and reduce the hepatic venous-portal gradient, a TIPS can in theory decrease the peri-operative risks attributable to portal hypertension. However, data are limited evaluating the benefit of preemptive TIPS placement on post-surgical outcomes. Several early case series and small retrospective cohort studies concluded that portal decompression via TIPS carries an acceptable risk and may enable select patients to undergo subsequent surgery; [101–103] these early studies also found no significant difference in post-operative complications or mortality following abdominal surgery between those who did and did not receive TIPS [104–106]. More recently, two single-center retrospective studies have suggested improved surgical outcomes for TIPS recipients. In a multivariable analysis, Piecha et al. found a lower incidence of inpatient mortality and liver transplantation for patients with cirrhosis who underwent TIPS compared to controls (19% vs. 40% for a composite outcome, respectively; *p* = 0.003) [107]. In a propensity-matched analysis, Chang et al. demonstrated that patients with cirrhosis undergoing surgery with pre-operative TIPS compared to those without TIPS had lower rates of acute-on-chronic liver failure (13% vs. 33% at 90-days; *p* = 0.020) and mortality (18% vs. 38% at 1-year; *p* = 0.023) [108]. In contrast, the largest retrospective study to date, Tang et al., found that recent TIPS placement among U.S. Veterans with cirrhosis who underwent major surgery was associated with increased risk of post-operative mortality (HR = 2.69, 95% CI: 1.37–5.30, *p* = 0.004) and worsened pre-operative liver synthetic function (lower albumin and higher INR, both *p* < 0.01) in propensity-matched analysis [109]. An important distinction of the latter study was that patients were matched prior to TIPS placement (as opposed to adjustment of covariates immediately prior to surgery); the authors demonstrated that TIPS patients developed lower albumin, higher INR, and higher bilirubin levels leading up to surgery, changes which were exacerbated in the post-operative period. This suggests that benefits of addressing portal hypertension through TIPS may be offset in part by worsened liver synthetic dysfunction. Because of these conflicting data, additional research is needed to evaluate prospectively the role for TIPS placement prior to surgery. Pre-operative TIPS should be considered on an individual basis at experienced centers, taking into account traditional indications for TIPS including refractory ascites and prevention of variceal bleeding [100]. Although clinical guidelines suggest no absolute MELD threshold to contraindicate TIPS [100], many centers consider patients with a MELD ≥ 15–18 at too high risk of mortality and liver dysfunction to proceed with TIPS placement [110, 111]. 

## Conclusion

Patients with cirrhosis are at an increased risk of post-operative mortality and complications due to underlying pathophysiologic changes of advanced liver disease, including portal hypertension, sarcopenia/malnutrition, and altered hemostasis. They require a careful pre-operative assessment with a detailed history, physical examination, laboratory testing, and imaging to identify and address individual risk factors for surgery. While several prediction models have been developed to aid pre-operative risk stratification, such as the CTP score, MELD score, MRS, and VPS, they should be used in conjunction with clinical judgement, taking into account patients’ severity of liver disease, medical comorbidities, and surgery-specific considerations. Prior to surgery, attempts should be made to optimize hepatic decompensations and other surgical risk factors, including addressing ascites, nutritional status, and/or physical deconditioning. Ultimately, the pre-operative assessment of patients with cirrhosis should be a comprehensive and individualized approach to improve outcomes and minimize complications following surgery.

## Key References

*Of major importance*

- Mahmud N, et al. Risk Prediction Models for Post-Operative Mortality in Patients With Cirrhosis. Hepatology. 2021;73(1):204-18.
-
  - This reference derives and internally validates the VOCAL-Penn Score to predict post-operative mortality in patients with cirrhosis.
- Teh SH, et al. Risk factors for mortality after surgery in patients with cirrhosis. Gastroenterology. 2007;132(4):1261-9.
-
  - This reference derives the Mayo Risk Score to predict post-operative mortality in patients with cirrhosis.

*Of importance*

- Mahmud N, et al. In-Hospital mortality varies by procedure type among cirrhosis surgery admissions. Liver Int. 2019;39(8):1394-9.
-
  - This reference highlights the variable risk of in-hospital mortality by surgical procedure category.
- Canillas L, et al. Comparison of Surgical Risk Scores in a European Cohort of Patients with Advanced Chronic Liver Disease. J Clin Med. 2023;12(18).
-
  - This reference compares the performance of cirrhosis surgical risk models in a European retrospective cohort with advanced chronic liver disease.
- Mahmud N, et al. Risk Prediction Models for Postoperative Decompensation and Infection in Patients With Cirrhosis: A Veterans Affairs Cohort Study. Clin Gastroenterol Hepatol. 2022;20(5):e1121-e34.
-
  - This reference evaluates the ability of surgical risk models to predict post-operative decompensation and infection.

## Data Availability

No datasets were generated or analysed during the current study.

## Abbreviations

ALFA: Cute liver failure
ACS: American College of Surgeons
ASA: American Society of Anesthesiologists
CTP: Child-Turcotte-Pugh
CSPH: Clinically significant portal hypertension
ERCP: Endoscopic retrograde cholangiopancreatography
GLP-1: Glucagon-like peptide-1
HPVG: Hepatic venous pressure gradient
INR: International normalized ratio
MRS: Mayo Risk Score
MASLD: Metabolic-associated steatotic liver disease
MELD: Model for End-Stage Liver Disease
NSQIP: National Surgical Quality Improvement Program
RYGB: Roux-en-y gastric bypass
TIPS: Transjugular intrahepatic portosystemic shunt
UNOS: United Network for Organ Sharing
U.S: United States
VPS: VOCAL-Penn Score
